# Identification of Type II Interferon Receptors in Geese: Gene Structure, Phylogenetic Analysis, and Expression Patterns

**DOI:** 10.1155/2015/537637

**Published:** 2015-08-06

**Authors:** Hao Zhou, Shun Chen, Yulin Qi, Qin Zhou, Mingshu Wang, Renyong Jia, Dekang Zhu, Mafeng Liu, Fei Liu, Xiaoyue Chen, Anchun Cheng

**Affiliations:** ^1^Institute of Preventive Veterinary Medicine, Sichuan Agricultural University, Chengdu, Sichuan 611130, China; ^2^Avian Disease Research Center, College of Veterinary Medicine of Sichuan Agricultural University, Chengdu, Sichuan 611130, China; ^3^Key Laboratory of Animal Disease and Human Health of Sichuan Province, Sichuan Agricultural University, Chengdu, Sichuan 611130, China

## Abstract

Interferon *γ* receptor 1 (IFNGR1) and IFNGR2 are two cell membrane molecules belonging to class II cytokines, which play important roles in the IFN-mediated antiviral signaling pathway. Here, goose IFNGR1 and IFNGR2 were cloned and identified for the first time. Tissue distribution analysis revealed that relatively high levels of goose IFN*γ* mRNA transcripts were detected in immune tissues, including the harderian gland, cecal tonsil, cecum, and thymus. Relatively high expression levels of both IFNGR1 and IFNGR2 were detected in the cecal tonsil, which implicated an important role of IFN*γ* in the secondary immune system of geese. No specific correlation between IFN*γ*, IFNGR1, and IFNGR2 expression levels was observed in the same tissues of healthy geese. IFN*γ* and its cognate receptors showed different expression profiles, although they appeared to maintain a relatively balanced state. Furthermore, the agonist R848 led to the upregulation of goose IFN*γ* but did not affect the expression of goose IFNGR1 or IFNGR2. In summary, trends in expression of goose IFN*γ* and its cognate receptors showed tissue specificity, as well as an age-related dependency. These findings may help us to better understand the age-related susceptibility to pathogens in birds.

## 1. Introduction

The interferon (IFN) *γ* cytokine can be induced by pathogens or artificial stimulation, which subsequently activates antiviral, antiproliferative, and immunomodulatory effects through recognizing specific receptors on the surface of target cells [1, 2]. The IFN*γ* receptor (IFNGR), a heterodimer consisting of two chains, IFNGR1 and IFNGR2, can be activated by IFN*γ* to transduce the downstream antiviral signal [3]. IFNGR1 and IFNGR2 are single transmembrane (TM) proteins belonging to the class II cytokine family, which likely function as the gateway to the control of IFN-mediated cellular signaling. As the ligand-binding subunit, IFNGR1 possesses an intracellular binding site for Janus tyrosine kinase (JAK) 1, a signal transducer and activator of transcription 1 (STAT1) [1]. The JAK2 binding site is located in an intracellular domain of IFNGR2, which serves as a signal-transducing subunit [1]. All of these sites are essential for the recruitment and activation of JAK1/JAK2 and subsequent phosphorylation of STAT1. The activated STAT1 homodimer then translocates to the nucleus and binds to the regulatory sequence (IFN*γ*-activated sequence) to promote gene transcription [4, 5]. Moreover, IFN*γ* can also regulate the antiviral gene transcription via IFN-stimulated gene factor 3 (ISGF3), thus inducing an effective immune response [6].

To date, studies have focused mainly on mammalian IFN*γ* systems, but little attention has been paid to avian IFN*γ* and its receptors. Chicken IFNGR1 was cloned from peripheral blood lymphocytes (PBLs) using the rapid amplification of cDNA ends (RACE), and the three-dimensional structure of its extracellular region was identified [7]. The extracellular region of chicken IFNGR2 also shares a similar structure with its human IFNGR counterpart [8]. In chickens, assessment of age-related expression of IFN, IFN receptors, and pattern recognition receptors (PRRs) has indicated that the IFN system is somewhat immature during the early developmental stage of chick embryonic cells [9]. The development of IFN*γ* in the intestinal immunity of juvenile chickens has been characterized as well [10].

Based on a comprehensive review of reports on the gene structure, evolutionary analysis, and crosstalk between IFN and its cognate receptors in birds [11], studies of the IFN system in waterfowl appear to be lagging behind. In addition, the development and immune characteristics of avian IFN*γ* are still poorly understood. Moreover, the duck IFNGR1 and IFNGR2 genes are only predicted sequences. Up to now, no information has been made available on the identification of goose IFN receptors. Given these considerations, this study was conducted to examine the expression level of goose IFN*γ* and its associated receptors throughout the embryogenesis phase and posthatch period. Herein, for the first time, goose IFNGR1 and IFNGR2 cDNA sequences were identified, and the corresponding amino acid sequences as well as structural characteristics were analyzed. Comparative analysis of goose IFNGR sequences with those in birds, mammals, fish, and reptiles may shed light on the evolutionary position of goose genes among vertebrates. The tissue distribution and age-related expression of goose IFN*γ* and IFN*γ* receptors also were analyzed in this study. The results of this study will extend existing information on the age-related development of goose IFN*γ* and its cognate receptors, which may shed further light on IFN antiviral responses in this species.

## 2. Methods

### 2.1. Animals

The study was conducted with Sichuan White Geese (Chinese goose,* A. cygnoides*). Goose embryos at 20 embryonic incubation days (EID20), goslings (1 week of age), and adult geese (3 months of age) were chosen. All animals in this study were purchased from the farm at Sichuan Agricultural University (Ya'an city, Sichuan province). One-week-old goslings and adult geese were maintained for 3 days in laboratory animal rooms for acclimation prior to experiments, and water and fodder were provided. The welfare of the animals was ensured during the sampling process.

### 2.2. RNA Extraction and cDNA Synthesis

The birds were euthanized, and then tissues were collected and snap-frozen in liquid N_2_. The chosen tissues included cecal tonsil, liver, lung, kidney, harderian gland, brain, bursa of Fabricius, cecum, heart, small intestine, spleen, thymus, gizzard, and proventriculus. Total RNA was extracted from various tissues using Trizol reagent (Invitrogen, Carlsbad, CA, USA) according to the manufacturer's instructions. The cDNA was synthesized using the QuantScript RT kit (Promega, Madison, WI, USA) according to the manufacturer's instructions. Finally, cDNA templates of all different samples were stored at −80°C until use.

### 2.3. Molecular Cloning of Goose IFNGR

A partial sequence of goose IFNGR was amplified by the degenerate primers F1, R1, F2, and R2 (all primer sequences used in this study are listed in Table 1), which were designed based on the conserved regions among its counterparts in birds (all reference sequences used in this study are listed in Table 2). The resultant PCR fragments were subcloned into the pGEM-T Easy Vector (Promega), followed by transformation of DH5*α* cells. The positive clones were sequenced by using the ABI 3730 XL sequencer (Applied Biosystems, Foster City, CA, USA). Subsequently, 3′ and 5′ rapid amplification of cDNA ends (RACE) was performed to obtain the full-length cDNA sequence of target genes. Based on the partial sequence obtained, Gene Specific Primers (GSPs), including 3GSP1, 3GSP2, 5GSP1, 5GSP2, and 5GSP3, were designed to obtain the full-length goose IFNGR cDNA. For 3′-RACE, the first strand cDNA was synthesized using the Adapter Primer (AP). The 3′-end of goose IFNGR was amplified by nested PCR using the primers 3GSP1 and 3GSP2 with AP1 and AP2. For 5′-RACE, the first strand cDNA was synthesized by using the primer 5GSP1 and M-MLV Reverse Transcriptase (Promega). A homopolymeric tail was then added to the 3′-end of the cDNA using TdT and dCTP (TaKaRa, Kyoto, Japan). The 5′-end of goose IFNGR was also obtained by two nested PCRs with the primer pairs 5GSP2/Abridged Anchor Primer (AAP) and 5GSP3/Abridged Universal Amplification Primer (AUAP). Finally, the full-length coding sequence of goose IFNGR was amplified by using Primer STAR Max DNA polymerase (TaKaRa).

### 2.4. Bioinformatic Analysis of Sequences

Potential open reading frames (ORFs) were analyzed by using the ORF finder program (http://www.ncbi.nlm.nih.gov/gorf/gorf.html) and translated into the corresponding amino acids using DNAMAN. N-Glycosylation sites were predicted with online software (http://www.cbs.dtu.dk/services/NetNGlyc/). Afterwards, the TM region was examined with the TMHMM server version 2.0 (http://www.cbs.dtu.dk/services/TMHMM/). The potential protein domains of amino acid sequences were forecasted via the SMART server (http://smart.embl-heidelberg.de/). Pairwise identity analysis was performed with the Species Demarcation Tool [12]. Alignment of putative amino acid sequences of IFNGR1 and IFNGR2 was performed using the Clustal program, and sequence similarities were calculated with the MegAlign program. Secondary structures were analyzed using the I-TASSER program (http://zhanglab.ccmb.med.umich.edu/). To analyze the evolutionary relationships between type II IFN receptors in birds and other vertebrates, a phylogenetic tree was constructed using amino acid sequences via the neighbor-joining (NJ) method in MEGA4 with bootstrap analysis based on 1000 repetitions [13].

### 2.5. Tissue Distribution and Age-Related Expression Analysis of Goose IFNGR mRNA

The tissue distribution of IFNGR in healthy 1-week-old goslings was studied by real-time quantitative qPCR (RT-qPCR) using the Bio-Rad CFX96 Real-Time Detection System. The age-related expression analysis of goose IFN*γ* receptors at the mRNA level in certain tissues of geese (embryonic incubation 20 days and adult) was also detected by RT-qPCR. Where possible, the primers were designed across intron and extron boundaries. Reactions were carried out in triplicate each in a total reaction volume of 10 *μ*L, including 0.8 *μ*L cDNA sample, 5 *μ*L SYBR Green PCR master mix (QuantiFast SYBR Green PCR Kit), 0.3 *μ*L of each primer (listed in Table 1), and 3.6 *μ*L ddH_2_O. The amplification program was 94°C for 4 min, followed by 40 cycles of 94°C for 10 s and 58°C for 30 s. After the amplification phase, a melting curve analysis (from 65°C to 95°C with a heating rate of 0.5°C per second and a continuous fluorescence measurement) was routinely performed to confirm the presence of a single and specific PCR product. Standard curves were generated for each gene from 10-fold serial dilutions of PCR products to estimate amplification efficiency. Finally, RT-qPCR data were analyzed by the 2^−ΔΔCT^ method using Bio-Rad CFX Manager Software.

### 2.6. Transcriptional Levels of IFN*γ* and IFNGR in Goose Mononuclear Cells (MNCs) after R848 Stimulation

Goose (3 months of age) spleen MNCs were collected, cultured in RPMI1640 (Gibco, Gaithersburg, MD, USA), and then seeded into 24-well cell culture plates in 10% serum-containing RPMI1640 medium. Thereafter, the cells were stimulated with R848 (20 *μ*g/mL) (Invivogen, San Diego, CA, USA) for 10 h, while PBS-treated cells were chosen as a control. IFN*γ* and IFNGR transcripts were detected by RT-qPCR according to methods described above.

## 3. Results

### 3.1. Sequence Analysis of Goose IFNGR1

**Figure 1:**
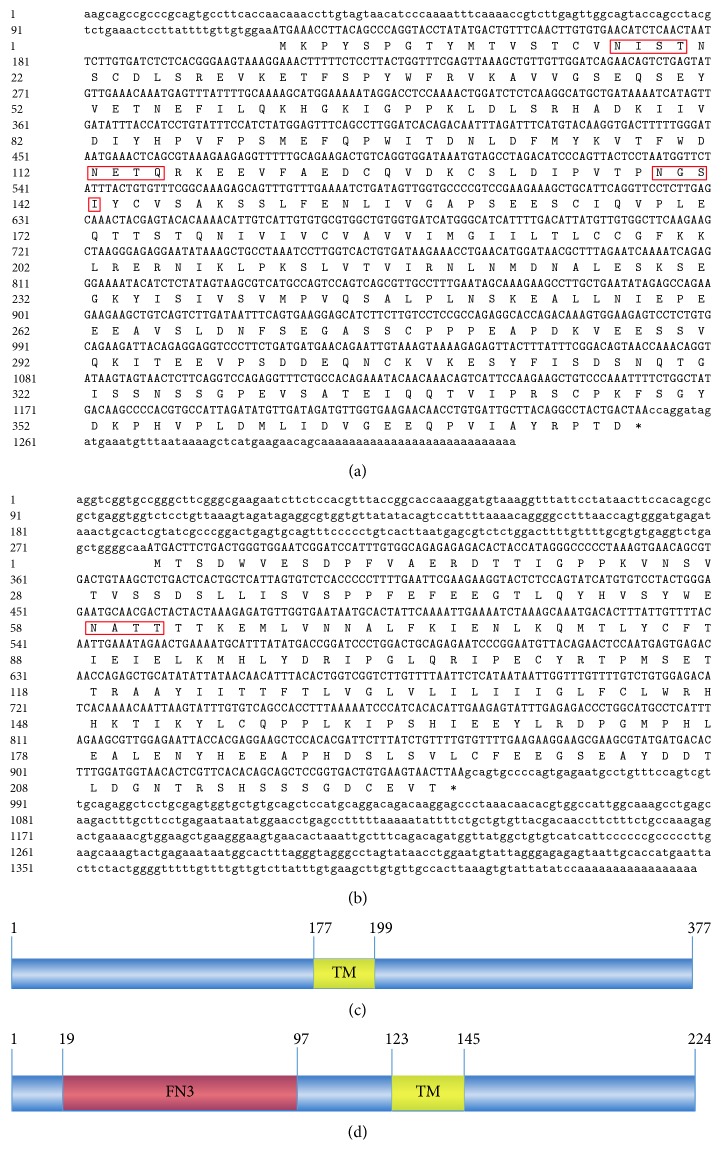
Nucleotide sequences of goose IFNGRs and deduced amino acid sequence structure. (a) Nucleotide sequence of goose IFNGR1 and the deduced amino acid sequence. The 5′-UTR and 3′-UTR sequences are shown in lowercase letters, while the ORF is presented in uppercase letters. The putative amino acid sequence is highlighted in blue and presented below the capital letters. Potential N-glycosylation sites are boxed. (b) Nucleotide sequence of goose IFNGR2 and deduced amino acid sequence. (c) Predicted protein domains characteristic of IFNGR1 and their alignment with counterparts from other birds and mammals. Conserved sequences are represented by the graph under the alignment. TM domains are marked in light yellow. (d) Predicted protein domains characteristic of IFNGR2. TM domains are marked in light yellow, while the fibronectin type III domain (FN3) is marked in light red.

The full-length (1322 bp) cDNA of goose IFNGR1 [GenBank: KM457284] contains a 117 bp 5′-UTR, a 1134 bp single open reading frame encoding 377 amino acids, and a 71 bp 3′-UTR (Figure 1). Three potential N-glycosylation sites were found in the goose IFNGR1 amino acid sequence (Figure 1). Only one TM domain was identified in goose IFNGR1, indicating that it is a single membrane protein (Figure 1).

**Figure 2:**
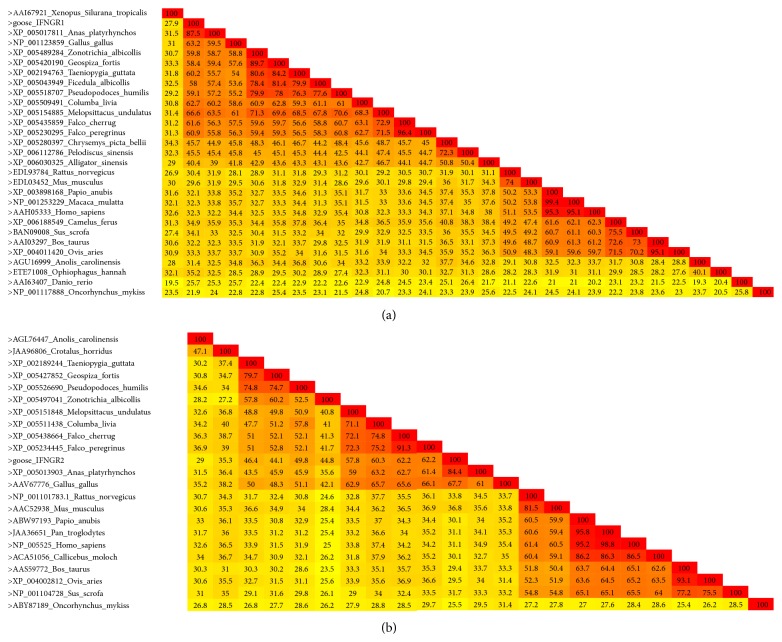
Heat map of IFNGR sequences in different species. The 2D color-coded matrix, decorated with a full color spectrum scheme, of IFNGR1 (a) and IFNGR2 (b) based on pairwise identity scores was constructed using the Species Demarcation Tool (STD).

Additionally, the deduced amino acid sequence of goose IFNGR1 was compared with those of avian and mammalian species. According to the 2D color-coded matrix generated based on a pairwise sequence alignment analysis (Figure 2), goose IFNGR1 shared the highest identity with its counterpart in* Anas platyrhynchos *[GenBank: XP005017811] (87.5%), which is much higher than that of* Homo sapiens *[GenBank: AAH05333] (32.3%) and* Danio rerio *[GenBank: AAI63407] (25.7%). Notably, the IFNGR1 amino acid sequence of* Gallus gallus *[GenBank: NP001123859] showed a lower identity with that of goose (63.2%) than that of duck (87.5%).

**Figure 3:**
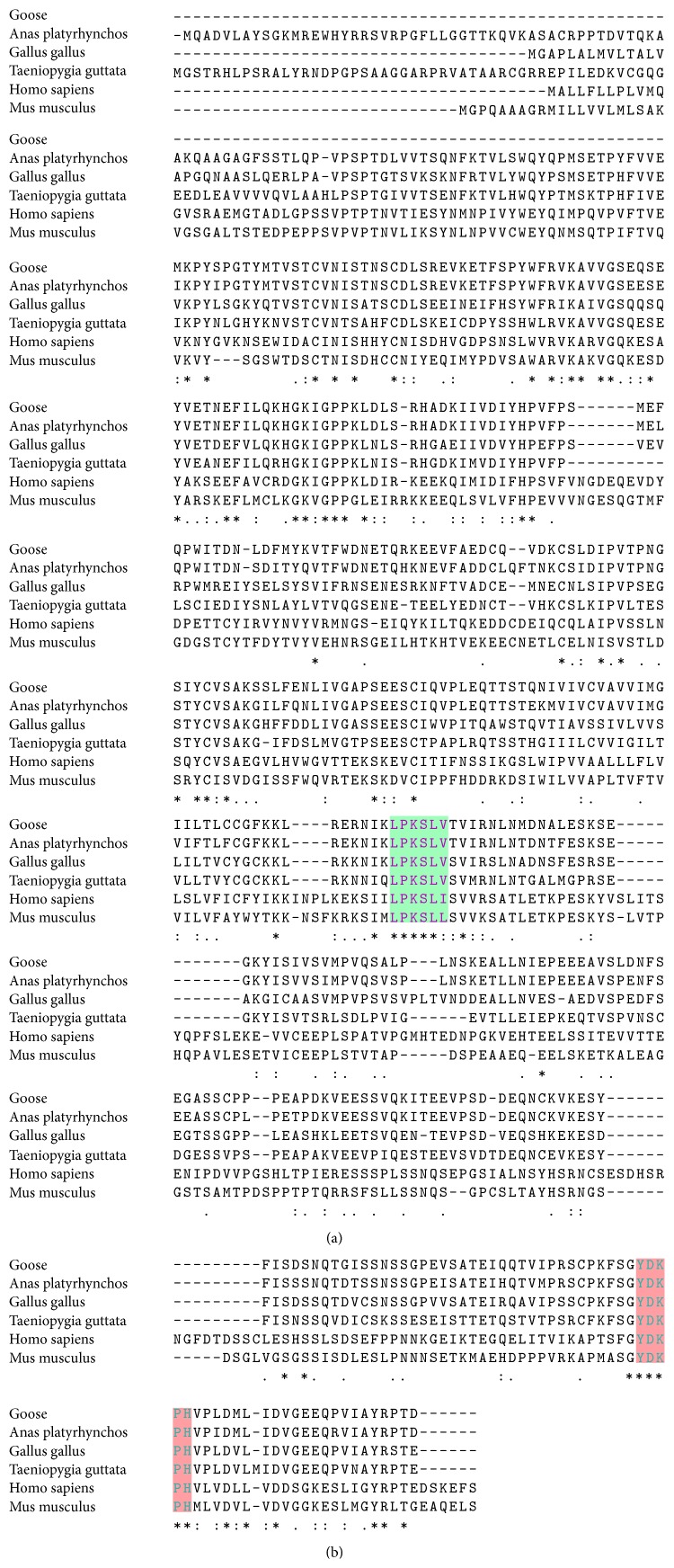
Multiple alignment analysis of IFNGR1 amino acid sequences from geese, birds, and mammalians. Selected species and GenBank accession numbers are as follows:* A. platyrhynchos* [XP005017811],* G. gallus* [NP001123859],* Taeniopygia guttata* [XP002194763],* H. sapiens *[AAH05333.1], and* M. musculus* [EDL03452.1]. The alignment was generated with ClustalW and modified manually. Amino acids conserved among all species are indicated as identical (*∗*), highly conserved (:), or weakly conserved (.). The light green shade highlights the JAK1 binding site, while the light red shade indicates the STAT1 binding site.

The multiple sequence alignment analysis showed that five cysteine sites and five tyrosine sites are completely conserved in birds and mammals (Figure 3). Furthermore, the JAK1 binding site (LPKSLV) and STAT1 binding site (YDKPH) were found in goose IFNGR1, which is highly similar to those of human and mouse (Figure 3).

### 3.2. Sequence Analysis of Goose IFNGR2

In this study, goose IFNGR2 was also cloned for the first time. The full-length cDNA of goose IFNGR2 [GenBank: KM461716] obtained was 1438 bp, with an open reading frame of 675 bp encoding for 224 amino acids (Figure 1). The 5′-UTR and 3′-UTR of IFNGR2 were 280 bp and 483 bp in length, respectively. IFNGR2 was predicted to have only one N-glycosylation site at the 58th amino acid (Figure 1). Unlike goose IFNGR1, goose IFNGR2 was found to have a TM domain and a fibronectin type III domain (FN3).

The color-coded matrix based on amino acid sequence alignment (Figure 2) showed that goose IFNGR2 shared the highest identity with* A. platyrhynchos *IFNGR2 [GenBank: XP005013903] (84.4%). Meanwhile, it shared 67.7% identity with* G. gallus* IFNGR2 [GenBank: AAV67776], 62.2% identity with* Falco cherrug *IFNGR2 [GenBank: XP005438664], and 60.3% identity with* Columba livia* IFNGR2 [GenBank: XP005511438].

**Figure 4:**
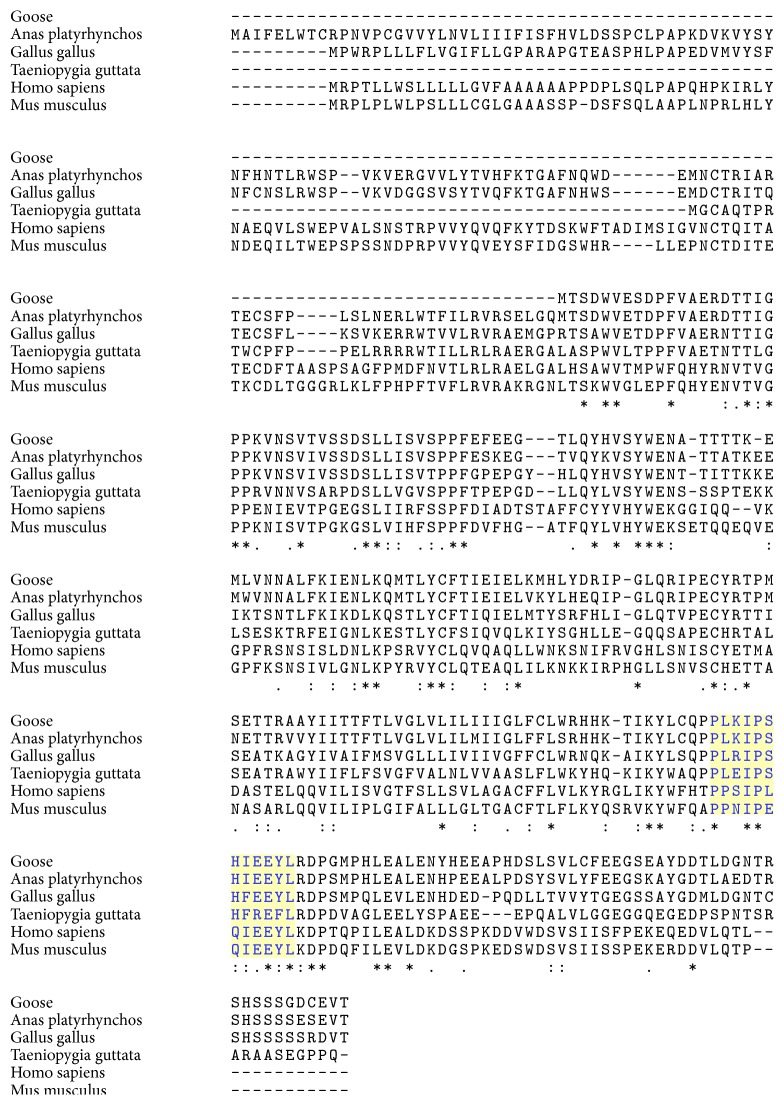
Multiple alignment analysis of IFNGR2 amino acid sequences from several birds and mammals. Selected species and GenBank accession numbers are as follows:* A. platyrhynchos* [XP005013903],* G. gallus* [AAV67776],* T. guttata* [XP002189244],* H. sapiens* [NP005525], and* M. musculus* [AAC52938]. The alignment was generated with ClustalW and modified manually. Amino acids conserved among all species are indicated as identical (*∗*), highly conserved (:), or weakly conserved (.). The light yellow shade highlights the JAK2 binding site.

The multiple sequence alignment analysis of IFNGR2 showed that two cysteine sites and four tyrosine sites were completely conserved in birds and mammals (Figure 4). Consistent with the human and mouse counterparts, goose IFNGR2 also had a JAK2 binding site (PLKIPSHIEEYL) located in a span from position of 158 to 169 (Figure 4).

### 3.3. Secondary Structural Model of Goose IFNGR1 and IFNGR2

**Figure 5:**
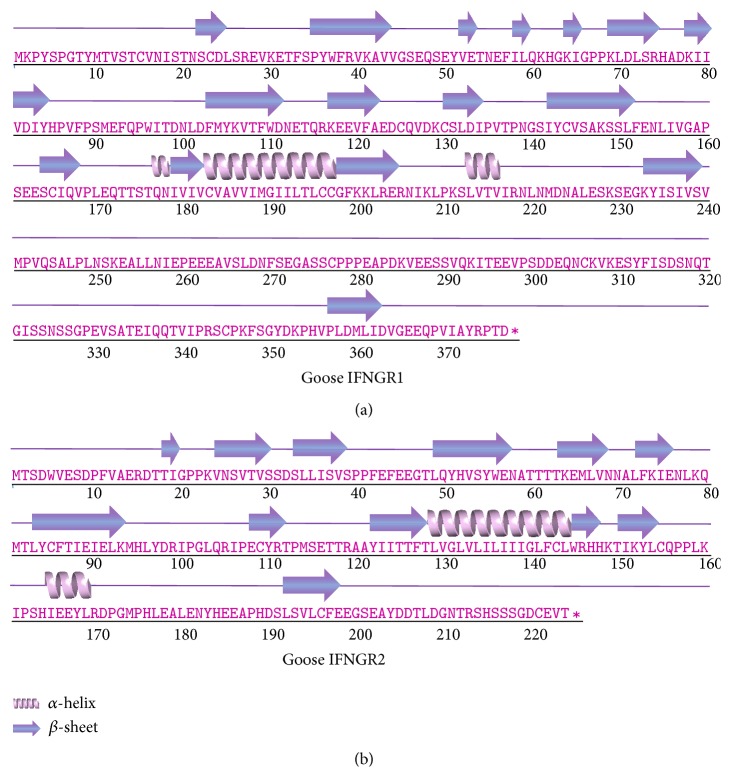
Secondary structures of goose IFNGR1 and IFNGR2. Secondary structures of goose IFNGR1 and IFNGR2 were analyzed using the I-TASSER online server. Both *α*-helices and *β*-sheets are shown in corresponding positions above the sequence.

As depicted in Figure 5, the secondary structure of goose IFNGR1 protein was predicted to contain 3 *α*-helices and 17 *β*-sheets. Meanwhile, the goose IFNGR2 amino acid sequence was predicted to contain 2 *α*-helices and 12 *β*-sheets. Although the IFNGR1 amino acid sequence was longer than that of IFNGR2, their secondary structures were observed to be similar.

### 3.4. Phylogenetic Analysis of Goose IFNGR

**Figure 6:**
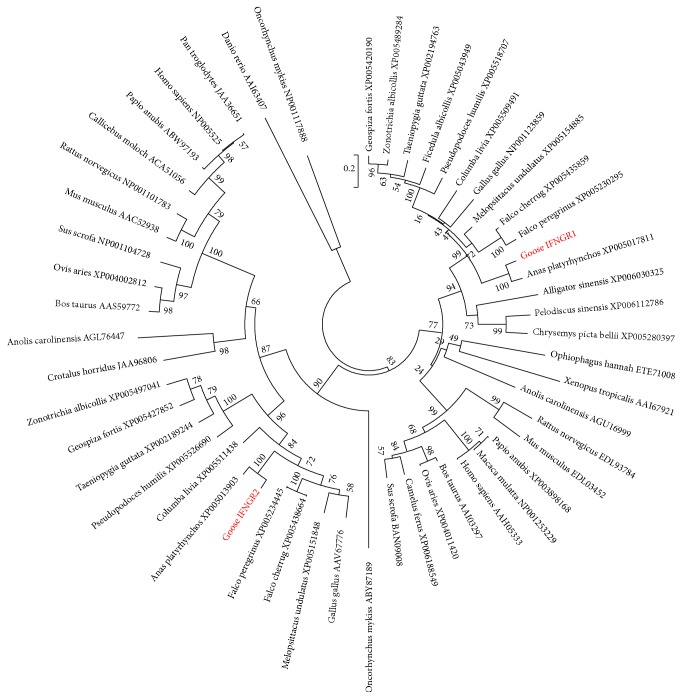
Phylogenetic analysis based on IFNGR1 and IFNGR2 amino acids. The phylogenetic tree of partial vertebrate IFNGR1 and IFNGR2 amino acid sequences was constructed using the NJ method in MEGA5. Numbers at branch nodes indicate the confidence level with 1000 bootstrap replications. IFNGR1 and IFNGR2 of birds are indicated with a green oval, and those of geese are indicated with red circles.

To clarify the evolutionary relationship between IFNGR of geese and other species, a phylogenetic tree was constructed with the amino acid sequences based on a Poisson model as shown in Figure 6. These sequences were mainly separated into four clusters of avian, mammalian, fish, and amphibian/reptilian groups. The phylogenetic analysis showed that the IFNGR1 and IFNGR2 clusters were divergent subgroups. Furthermore, goose IFNGR1 appeared to be closely related to its counterparts among birds, especially duck IFNGR1. Analysis of the bird group also revealed that the goose IFNGR1 and duck IFNGR1 sequences were located in the same monophyletic group, which was distinct from other birds, such as chickens, pigeons, and sparrows. Similar results also were observed with goose IFNGR2. Furthermore, the genetic distance of fish sequences analyzed was relatively far from those of avian species, and goose IFNGR1 and IFNGR2 showed the farthest distance from the fish IFNGR molecules.

### 3.5. Tissue Distribution of Goose IFN*γ* and IFNGR

**Figure 7:**
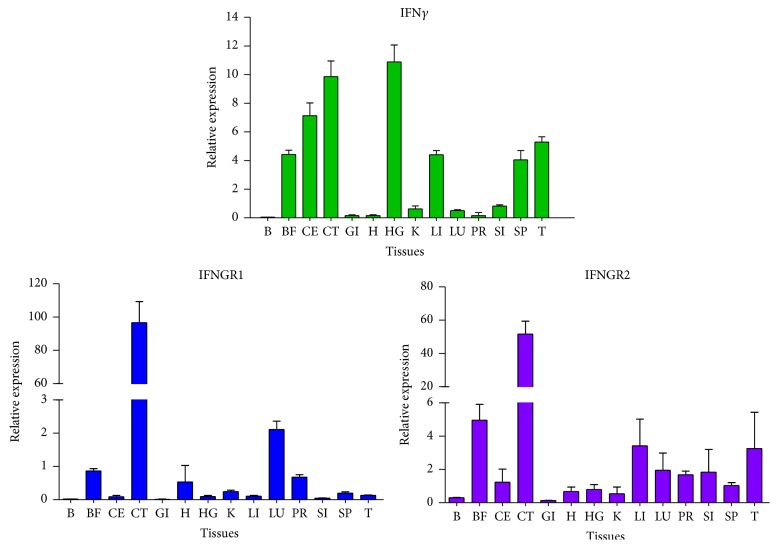
Tissue distribution of IFN*γ*, IFNGR1, and IFNGR2 in goslings. Tissues of three goslings (1 week of age) were collected, and mRNA levels of IFN*γ*, IFNGR1, and IFNGR2 (normalized to *β*-actin) were quantified by RT-qPCR. Data are represented as the mean ± SEM (*n* = 3). Cecal tonsil: CT, liver: Li, lung: Lu, kidney: K, harderian gland: HG, brain: B, bursa of Fabricius: BF, cecum: CE, heart: H, small intestine: SI, spleen: Sp, thymus: T, gizzard: Gi, and proventriculus: Pr.

The quantitative analysis showed that the relative expression levels of IFN*γ*, IFNGR1, and IFNGR2 mRNA varied in different tested tissues (Figure 7). Relatively high levels of IFN*γ* were detected in the harderian gland, cecal tonsil, and cecum, followed by thymus, liver, bursa of Fabricius, and spleen, and the IFN*γ* expression was lowest in the brain. The goose IFNGR1 gene was highly expressed in the cecal tonsil, moderately expressed in the lung, bursa of Fabricius, heart, and proventriculus, and minimally expressed in the brain and gizzard. In addition, goose IFNGR2 was strongly detected in the immune-associated tissues, especially in the cecal tonsil and bursa of Fabricius. In most immune-related tissues, the relative mRNA transcriptional levels of IFN*γ*, IFNGR1, and IFNGR2 were similar at the same time point, and the ubiquitous expression of these genes in immune tissues of healthy goslings was observed.

### 3.6. Age-Related Expression Analysis of Goose IFN*γ* and IFNGR

**Figure 8:**
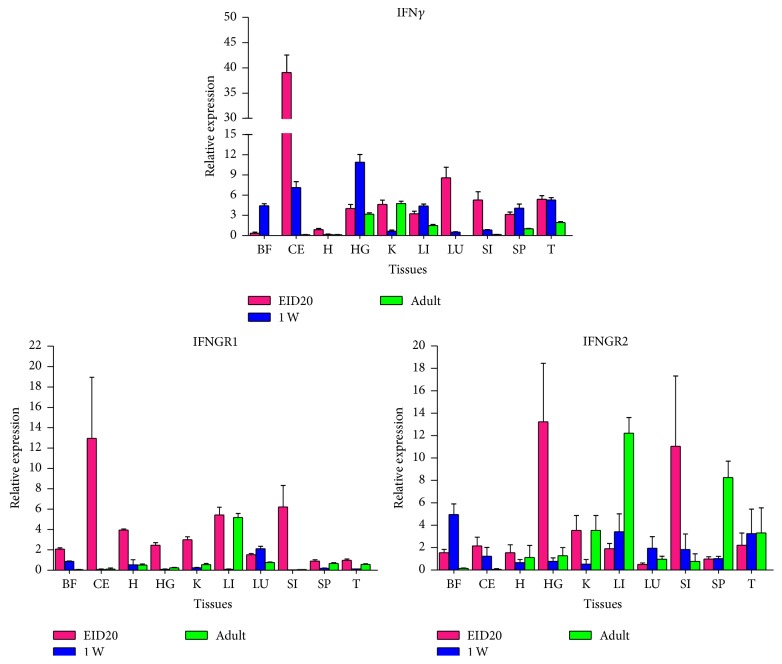
Age-related mRNA expression analysis of goose IFN*γ* and IFNGRs. Comparative mRNA sequence analysis of goose IFN*γ* and its receptors in certain tissues of embryos at EID20, goslings (1 week of age), and adult geese (3 months of age). *β*-actin was amplified as an internal control. Data are represented as the mean ± SEM (*n* = 3). Spleen: Sp, thymus: T, bursa of Fabricius: BF, harderian gland: HG, small intestine: SI, heart: H, liver: Li, lung: Lu, kidney: K, and brain: B.

To understand the expression patterns of IFN*γ* and its receptors, their mRNA levels in ten tissues of goose embryos, goslings, and adult geese were assessed by RT-qPCR (Figure 8). In goose embryos, the highest level of IFN*γ* was found in the cecum, while it was barely expressed in the brain. Meanwhile, IFNGR1 was detected at high levels in the cecum, small intestine, and liver and at lower levels in the heart, kidney, harderian gland, and bursa of Fabricius. In the embryonic stage, goose IFNGR2 was strongly transcribed in the harderian gland and small intestine. In the adult goose, IFN*γ* was strongly detected in the kidney and harderian gland. The highest level of IFNGR1 was seen in the liver, while IFNGR2 was strongly transcribed in the liver and spleen. However, no significant differences were observed in the expression of IFNGR2 in the heart, lung, and thymus.

Obvious decreases in IFN*γ* expression were observed in the cecum, small intestine, and lung during goose development. Notably, in the cecum, heart, harderian gland, kidney, liver, and small intestine, the transcriptional level of IFNGR1 in 1-week-old goslings was obviously lower compared with that in goose embryos. Furthermore, in the liver and spleen, the IFNGR2 transcriptional level was obviously increased, while it was apparently decreased in the small intestine and harderian gland.

### 3.7. Effect of R848 on Transcriptional Levels of Goose IFN*γ* and IFNGRs

**Figure 9:**
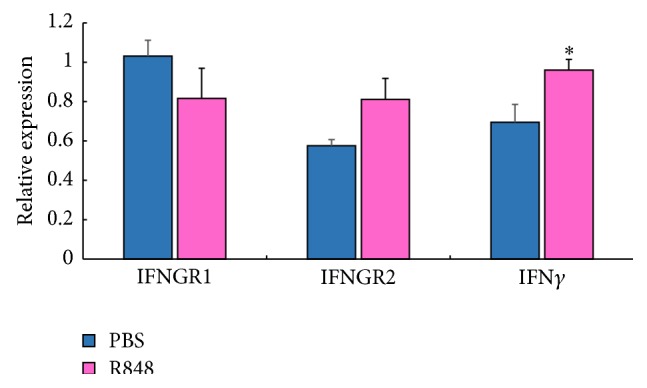
Effect of R848 on transcriptional levels of goose IFN*γ* and IFNGRs. The relative mRNA levels of IFNGR1, IFNGR2, and IFN*γ* at 10 h after stimulation of goose MNCs with R848. Each mRNA expression value was normalized by *β*-actin. Data are presented as the mean ± SEM (*n* = 4), and differences between agonist-treated cells and mock-treated cells were analyzed by the two-tailed *t*-test. ^*∗*^ *P* < 0.05.

As shown in Figure 9, R848 caused a highly significant upregulation of goose IFN*γ* (*P* < 0.05) compared to the PBS control, but no significant change in expression of IFNGR1 (*P* = 0.25) and IFNGR2 (*P* = 0.07) was detected. These results indicated that the R848 agonist could activate IFN*γ* but did not affect the expression of IFNGR1 or IFNGR2 in geese. The results above may facilitate further studies of the goose IFNGR-mediated immunological signaling pathway.

## 4. Discussion

IFN*γ* is a pleiotropic cytokine secreted by T-helper-1 (Th1) cells, promoting both innate and adaptive responses to infection within the host [14, 15]. The major producers of this cytokine are activated T cells, natural killer (NK) cells, and professional antigen-presenting cells (APCs) [16–18]. IFN*γ* binds to constitutively expressed IFN*γ* receptors, a heterodimer consisting of two chains, IFNGR1 and IFNGR2, which then activates the downstream JAK-STAT signaling pathway. The phosphorylated STAT proteins move into the nucleus, bind specific DNA response elements, and directly transcribe IFN-stimulated genes to induce an antiviral immune response. As reviewed elsewhere [19], IFN*γ* can contribute to the protection against infection with some viruses, such as hepatitis B virus, herpes simplex virus, and lymphocytic choriomeningitis virus. The antiviral responses may rely on the expression levels of IFNGR1 and IFNGR2, as well as the interaction between IFNGR and IFN*γ*.

Until now, comparatively little was known about avian IFN*γ* receptors at the molecular level other than those of chickens. Herein, we described the molecular cloning of goose IFNGR1 (1322 bp) and IFNGR2 (1438 bp) cDNA for the first time. Goose IFNGR1 and IFNGR2 were found to both possess a TM region, which demonstrated that they are single membrane proteins. The JAK1 binding site (positions 209–214) and STAT1 binding site (positions 351–355) of IFNGR1 were localized to the intracellular region, which can recruit JAK1 and STAT1 for signal transduction. The amino acids of these binding sites in birds have been reported to be relatively conserved in both humans and mice [3]. Similarly, the JAK2 binding site (positions 158–169) of IFNGR2 was also located at the intracellular region. These specific motifs are relatively conservative between birds and mammals [3].

In this study, the goose IFNGR1 and IFNGR2 amino acid sequences were analyzed at the structural and phylogenetic levels. Prior to this study, the secondary structures of IFN receptors of geese were largely unknown. We found that the secondary structure of the goose IFNGR1 protein contained 5.6%  *α*-helices, 23.6%  *β*-sheets, and 70.8%  random coils, which was different from that predicted for the counterpart protein in chickens [7]. Additionally, the goose IFNGR2 protein contained 9.8%  *α*-helices, 28.6%  *β*-sheets, and 61.6%  random coils, suggesting certain differences between the secondary structure of this protein in geese and chickens [8]. These results may aid in clarifying the tertiary structures of goose IFNGR1 and IFNGR2. Differences in secondary structures between IFNGR1 and IFNGR2 may result in subtle changes of the higher order structures and endow them with different functions. Additionally, the minimal divergence of IFNGR between geese and ducks further indicated the conservation of goose IFNGR1 and IFNGR2 during the evolution of waterfowl. The structural and evolutionary approaches to studying immune genes such as IFNGR will also help us to unravel interspecies similarities and differences in host defense.

Analysis of the tissue distribution of IFN*γ*, IFNGR1, and IFNGR2 in goslings showed that these genes were constitutively and widely expressed in different tissues. Notably, the results showed that expression patterns of IFN*γ*, IFNGR1, and IFNGR2 were not completely the same in different periods of development. IFN*γ* was widely detected in various samples, but the level of IFN*γ* in the brain of goslings was lowest. Similarly, IFNGR1 and IFNGR2 were found at relatively low levels in the brain. The main reason for these observations may be that the brain does not participate in the IFN-mediated immune response or lacks immune cells. Chickens infected with infectious bursal disease virus have shown extensive viral replication in the bursa and cecal tonsils with an associated accumulation of T cells [20]. In this study, both IFNGR1 and IFNGR2 were readily detected in the cecal tonsil of goslings. A possible explanation for this phenomenon is that abundant lymphocytes accumulate in the cecal tonsil, which is responsible for the intestinal antiviral immune response. The abundant expression of goose IFNGR1 and IFNGR2 in the cecal tonsil may contribute to the strong intestinal mucosal immunity. Notably, as shown in Figure 7, IFNGR1 and IFNGR2 levels in the lung were also relatively higher than those in the kidney and heart, which may be attributed to alveolar macrophages as being the predominant cells in the lung. As a result, the lungs can secrete a large number of bioactive cytokines, which subsequently participate in the mucosal immune defense. In addition, IFN*γ* and IFNGR2 were observed to be widely expressed in the immune-related tissues including the bursa of Fabricius, cecum, spleen, and thymus, while IFNGR1 was extensively expressed in the bursa of Fabricius and cecal tonsil. The similar tissue distribution of goose IFN*γ* and its cognate receptors suggested that these cytokines are immune-associated factors. To some extent, the induction of the IFN*γ* immune response may be reasonably connected with its associated receptors due to the similar tissue-specific expression patterns.

In order to explore the expression patterns during goose developmental period, we detected levels of IFN*γ*, IFNGR1, and IFNGR2 in goose embryos and adult geese. In the spleen, the expression of IFN*γ* increased from embryos to gosling during the early developmental period, which is consistent with prior observations of chicken IFN*γ* [21]. The decrease of IFN*γ* was observed in adult geese. One of the possible reasons for the reduction of IFN*γ* may be the functional degeneration of the spleen in adult geese. The expression of IFNGR2 also showed a downward trend in the spleen, while that of IFNGR1 did not, which differed from expression patterns of these genes in the harderian gland. These genes were expressed in an organ-specific manner, which was similar to the concept of tissue-specific innate immune gene expression profiles [21–24]. As both IFNGR1 and IFNGR2 are potentially TM proteins, their expression patterns remained stable in most tissues. Finally, the defective production of IFN*γ* may be compensated by the high expression of IFNGR2 in the adult period of development, thus keeping a certain balance of the effectiveness of IFN*γ* in the host defense system. However, in the cecum and small intestine, IFN*γ*, IFNGR1, and IFNGR2 were detected at extremely high levels during the embryonic stage, but they declined gradually during goose development. Previous studies had demonstrated that IFN*γ* directly affected the barrier function in model intestinal epithelial monolayers [25]. Receptors for IFN*γ* have been reported on the surface of epithelial cells [26] and endothelial cells [27]. Thus, observing high expression levels of goose IFN*γ* and its associated receptors in the cecum and small intestine in this study was reasonable. The results also indicated that the IFN*γ* immune system may be established during the embryonic stage. Furthermore, under unstimulated conditions, no specific correlation between the expression patterns of IFN*γ*, IFNGR1, and IFNGR2 in the same tissue was observed. Intriguingly, low expression of IFNGR1 in goslings may have been compensated by IFN*γ* and IFNGR2. These differences in the expression patterns of IFN*γ* and its receptors in geese to some extent may have been simply caused by the functional compensation of these molecules in different organs. Furthermore, R848 could significantly upregulate IFN*γ*, but it did not influence the expression of IFNGR1 and IFNGR2 by 10 h after stimulation. Altogether, these findings will expand our knowledge of IFNGR-mediated immune responses in waterfowl.

## 5. Conclusion

In summary, we have identified and characterized IFN*γ* receptors in geese for the first time, providing new insights into these immune molecules in this species. Goose IFN*γ* and its receptors were found to be transcribed primarily in immune-related tissues, but the overall age-related expression of goose IFN*γ*, IFNGR1, and IFNGR2 did not appear to be directly correlated. Furthermore, R848 could significantly induce IFN*γ* but not IFNGR1 or IFNGR2. Nevertheless, much work is still needed to clarify the interaction between goose IFN*γ* and IFNGR1 or IFNGR2, which will contribute to a better understanding of the antiviral defense system of aquatic birds.

## Figures and Tables

**Table 1:** List of primers and sequences.

Methods	Gene name	Primer name	Nucleotide sequence (5′-3′)
Reverse transcription		Oligo(dT)18	TTTTTTTTTTTTTTTTTT

Partial sequence	*IFNGR1 *	F1	TTAAAGCTGTTGTTGGATCA
		R1	CAATCACASGYTGTTCTTC
	*IFNGR2 *	F2	CTGAGGTGGTCTCCTGTTA
		R2	TCAAATACTCTTCAAWGTGTG

3RACE		AP	CCAGTGAGCAGAGTGACGAGGACTCGAGCTCAAGC (T)18
		AP1	CCAGTGAGCAGAGTGACG
		AP2	GAGGACTCGAGCTCAAGC

5RACE		AAP	GGCCACGCGTCGACTACGGGIIGGGIIGGGIIGGGIIG
		AUAP	GGCCACGCGTCGACTAGTAC

3RACE-GSP	*IFNGR1 *	G1-3GSP1	GGCACCAGACAAAGTGGAAGAGTC
		G1-3GSP2	TGCAGAAGATTACAGAGGAGGTCC
	*IFNGR2 *	G2-3GSP1	TGGACTGCGGAGAATCCCGGAATG
		G2-3GSP2	CAATGAGTGAGACAACCAGAGCTG

5RACE-GSP	*IFNGR1 *	G1-5GSP1	ATCCCAAAAAGTCACC
		G1-5GSP2	GAAATACAGGATGGTAAATATCAAC
		G1-5GSP3	GAGAGATCCAGTTTTGGAGGTC
	*IFNGR2 *	G2-5GSP1	CATTCTCCCAGTAG
		G2-5GSP2	AGTCACGCTGTTCACTTTAGGG
		G2-5GSP3	ATTCCACCCAGTCAGAAGTCAT

Real-time PCR	*IFNGR1 *	goqRT-G1-F	GCATTCAGGTTCCTCTTG
		goqRT-G1-R	AAGCGTTATCCATGTTCAG
	*IFNGR2 *	goqRT-G2-F	AATCTTCTCCACGTTTACCG
		goqRT-G2-R	CAGTAGAAGTAATTCATGGTG
	*β-actin *	goqRT-*β*actin-F	TCCCTGGAGAAGAGCTACGA
		goqRT-*β*actin-R	GTGTTGGCGTACAGGTCCTT

Degenerate bases: Y = C + T; W = A + T; and S = C + G.

**Table 2:** List of reference sequences.

Gene name	Organism	GenBank accession number
*IFNGR1 *	*Ficedula albicollis *	XM005043892
	*Taeniopygia guttata *	XM002194727
	*Gallus gallus *	NM001130387
	*Anas platyrhynchos *	XM005017754

*IFNGR2 *	*Ficedula albicollis *	XM005037096
	*Taeniopygia guttata *	XM002189208
	*Gallus gallus *	AY820753
	*Anas platyrhynchos *	XM005013846
